# Supporting successful implementation of public health interventions: protocol for a realist synthesis

**DOI:** 10.1186/s13643-016-0229-1

**Published:** 2016-04-07

**Authors:** Marjorie MacDonald, Bernadette Pauly, Geoff Wong, Kara Schick-Makaroff, Thea van Roode, Heather Wilson Strosher, Anita Kothari, Ruta Valaitis, Heather Manson, Warren O’Briain, Simon Carroll, Victoria Lee, Samantha Tong, Karen Dickenson Smith, Megan Ward

**Affiliations:** School of Nursing, University of Victoria, Room A402a, HSD Building, 3800 Finnerty Road, Victoria, BC V8P 5C2 Canada; Centre for Addictions Research of BC, Box 1700 STN CSC, Victoria, BC V8W 2Y2 Canada; Nuffield Department of Primary Care Health Sciences, University of Oxford, 2nd Floor, New Radcliffe House, Woodstock Road, Oxford, OX2 6GG UK; Faculty of Nursing, 5-276 Edmonton Clinic Health Academy, University of Alberta, 11405-87 Ave, Edmonton, AB T6R 1C9 Canada; Faculty of Human and Social Development, University of Victoria, Victoria, BC V8P 5C2 Canada; Joint Graduate Program in Health Information Science, CIHR New Investigator, School of Health Studies, University of Western Ontario, Health Sciences Building, room 222, London, Ontario Canada; School of Nursing, 3N25E, Health Sciences Centre, McMaster University, 1280 Main Street West, Hamilton, ON L8S 4K1 Canada; Health Promotion, Chronic Disease and Injury Prevention, Public Health Ontario | Santé publique Ontario, Suite 300, 480 University Avenue, Toronto, ON M5G 1V2 Canada; Public Health Services, British Columbia Ministry of Health, PO Box 9646, STN PROV GOVT, Victoria, BC V8W9P1 Canada; Department of Sociology, University of Victoria, PO Box 1700 STN CSC, Victoria, BC V8W 2Y2 Canada; Fraser Health Authority, Suite 400, Central City Tower, 13450 – 102nd Avenue, Surrey, BC V3T 0H1 Canada; Health Equity & Population Health Unit, Population and Public Health, Fraser Health Authority, 13450 – 102nd Ave, Surrey, BC V3T 0H1 Canada; Clinical Programs, Population & Public Health, Fraser Health Authority, Suite 400, Central City Tower, 13450 – 102nd Avenue, Surrey, BC V3T 0H1 Canada; Region of Peel Public Health, 44 Peel Centre Drive, 4th flr, Brampton, ON L6T 4B5 Canada

**Keywords:** Realist synthesis, Realist review, Public health, Population health, Implementation, Public health interventions, Knowledge translation

## Abstract

**Background:**

There is a growing emphasis in public health on the importance of evidence-based interventions to improve population health and reduce health inequities. Equally important is the need for knowledge about how to implement these interventions successfully. Yet, a gap remains between the development of evidence-based public health interventions and their successful implementation. Conventional systematic reviews have been conducted on effective implementation in health care, but few in public health, so their relevance to public health is unclear. In most reviews, stringent inclusion criteria have excluded entire bodies of evidence that may be relevant for policy makers, program planners, and practitioners to understand implementation in the unique public health context. Realist synthesis is a theory-driven methodology that draws on diverse data from different study designs to explain how and why observed outcomes occur in different contexts and thus may be more appropriate for public health.

**Methods:**

This paper presents a realist review protocol to answer the research question: Why are some public health interventions successfully implemented and others not? Based on a review of implementation theories and frameworks, we developed an initial program theory, adapted for public health from the Consolidated Framework for Implementation Research, to explain the implementation outcomes of public health interventions within particular contexts. This will guide us through the review process, which comprises eight iterative steps based on established realist review guidelines and quality standards. We aim to refine this initial theory into a ‘final’ realist program theory that explains important context-mechanism-outcome configurations in the successful implementation of public health interventions.

**Discussion:**

Developing new public health interventions is costly and policy windows that support their implementation can be short lived. Ineffective implementation wastes scarce resources and is neither affordable nor sustainable. Public health interventions that are not implemented will not have their intended effects on improving population health and promoting health equity. This synthesis will provide evidence to support effective implementation of public health interventions taking into account the variable context of interventions. A series of knowledge translation products specific to the needs of knowledge users will be developed to provide implementation support.

**Systematic review registration:**

PROSPERO CRD42015030052

**Electronic supplementary material:**

The online version of this article (doi:10.1186/s13643-016-0229-1) contains supplementary material, which is available to authorized users.

## Background

Implementation is ‘the Achilles heel of innovation’ ([1], p. 10) and is often defined as an evidence-to-practice gap [2–5] in which successful implementation of evidence-based interventions is fraught with challenges [4, 5]. If public health program and policy interventions are not implemented effectively, they will not have their intended effects on improving population health or reducing health inequities. Furthermore, the cost to the system will be considerable in a time of scarce public health resources [6, 7]. Given opportunity costs, a poorly implemented intervention can quickly erode policy and practice support, creating more challenges to ‘getting it right’ over the longer term [8].

Implementation research, often equated with knowledge translation [9, 10], has been conducted across many disciplines [1, 11] to document the frequency of unsuccessful implementation of policies and programs, identify factors influencing successful implementation, evaluate the effectiveness of implementation strategies, and develop theoretical frameworks to analyze or guide the implementation process. Systematic reviews of implementation studies have been conducted in several fields [12–15], but when we began this project, we had not located any comprehensive systematic reviews of implementation specific to public health. Because of public health’s population focus and location in the community, there may be unique features of public health systems and interventions that raise questions about the applicability of the broader health care implementation literature to public health interventions [16, 17].

The UK’s National Institute for Clinical Excellence, in their work to develop public health guidance [17], identified three problems with Cochrane-style systematic reviews for public health: (1) the breadth of the public health evidence base is vast, encompassing social, political, economic, and cultural factors; (2) explanations of effects in public health are multi-level; and (3) the length of the causal chain in public health interventions is extended, not proximal or direct as in clinical interventions. These factors make it very difficult to utilize randomized controlled experimental designs in the study of public health interventions.

To elaborate, public health has an expansive interdisciplinary evidence base that draws on diverse data types as well as on social science theories and methodologies that do not make it to the top of most evidence hierarchies (e.g., Cochrane), despite their legitimacy in many other disciplines. This proposition was supported by Kelly et al. [17] who found that search strategies and inclusion criteria in systematic reviews immediately eliminated significant bodies of evidence. For example, one review on knowledge translation strategies in public health located 346 potentially relevant publications but only five met the inclusion criteria [18] thereby excluding many articles that could provide relevant guidance on implementation to public health decision makers. Others have argued similarly that conventional systematic reviews are often not relevant in public health because there may be ethical constraints in randomly selecting or assigning people to experimental conditions [19].

An emerging methodological alternative is realist review or synthesis [20–23], which may be more helpful to knowledge users [21, 22] in public health for synthesizing evidence on effective implementation [23–26]. Realist reviews are now widely accepted in the field of research synthesis and are increasingly being published in journals like Systematic Reviews and Implementation Science.

Realist review allows for inclusion of a broad range of study designs with both qualitative and quantitative data. It is distinguished from other reviews by its focus on causal mechanisms in interaction with context to produce outcomes. In contrast to conventional reviews that focus on intervention effectiveness, realist synthesis is a theory-driven approach that aims to explain how and why observed outcomes occur. They focus explicitly on what works, for whom, and in what contexts. As a theory-driven approach, there are at least three levels of theory involved. A realist synthesis begins with an initial or ‘rough program theory’ [26] which is a general theory of the intervention(s) or program that lays out what is being investigated and how it is expected to work. It is not specified in realist terms—that is, with respect to contexts, mechanisms, and outcomes. The initial program theory guides the search, selection, and synthesis process but is continually refined throughout the review to create a realist program theory that specifies the relevant contexts, mechanisms, and outcomes and their configurations. Ultimately, the refined realist program theory is finalized as a middle-range theory. In realist synthesis, this is defined as a theory that is ‘detailed enough and close enough to the data that testable hypotheses can be derived from it but abstracted enough to apply to other situations’ ([26], p. 12).

Realism is the philosophy of science underlying realist synthesis. Pawson states that realism is: ‘…a methodological orientation, or a broad logic of inquiry that is grounded in the philosophy of science and social science’ [22]. Realists acknowledge the existence of an external reality that has an influence on human action. The notion of ‘mechanism’ is thus central in realism for explaining the relationship between the social world (context) and human actions or behaviour (outcomes) [27]. Realists argue that mechanisms have generative causation, or causal force. In realism, mechanisms can be defined as

… underlying entities, processes, or [social] structures which operate in particular contexts to generate outcomes of interest. Here ‘entities’ may refer to things such as norms or belief systems, ‘processes’ are sequences where later events depend on earlier ones, and social structures may refer to things such as gender, class, or cultural patterns of relationships ([26], p. 5).

The assumption behind realist synthesis is that an intervention will trigger mechanisms differently in different contexts (e.g., in different health authority or health unit organizational structures) to produce different outcomes (e.g., variable degrees of success in implementation [27]). In synthesizing the evidence, we seek to explain the interrelationships among context (C), mechanism (M), and outcomes (O) (i.e., CMO configurations or CMOCs). The locus of comparison across interventions is the mechanism(s), which may or may not be activated in particular contexts, and may or may not lead to the projected outcomes. The central question in realist synthesis is, What are the mechanisms that cause desired outcomes to occur and in what contexts are they triggered [26]?

The need for a realist synthesis of the research on implementation of public health interventions was identified by our research team comprising researchers and knowledge users (i.e., public health decision makers and practitioners) across two provinces. This team came together in 2007 to develop a program of research focussed on studying the implementation and impact of public health renewal policies in both provinces. Specifically, these policy interventions were the British Columbia Core Public Health Functions Framework [28] and the Ontario Public Health Standards [29]. Our findings in one study [1] suggested variable implementation within and between provinces influenced by unique contextual factors. As such, our team identified the need to undertake a realist synthesis that would provide knowledge users with timely, relevant, and usable information to guide implementation of subsequent public health policy and program interventions. This paper presents the protocol for our realist review.

## Method

### Study purpose

The purpose of this study is to conduct a realist synthesis of research on effective strategies to support implementation of public health interventions. Most public health interventions have the aim, explicitly or implicitly, to improve population health and/or promote health equity. The original overarching research question is, Why are some public health interventions successfully implemented and others not? To answer this question, we will search and assess relevant literature, develop a realist program theory on the implementation of public health interventions using an integrated knowledge translation process, and generate insights into the strategies for effective implementation.

### Objectives

The objectives of our study are to

1. Understand the contexts and mechanisms that influence the degree to which system-wide public health policies and programs are implemented.
2. Determine whether current implementation frameworks are adequate for public health at the population level.
3. Contribute to the development of the realist review methodology for public health interventions.
4. Develop a series of knowledge translation products that will be helpful to our knowledge user partners in supporting implementation of public health interventions in their organizations and beyond.

### Research plan

**Fig. 1:**
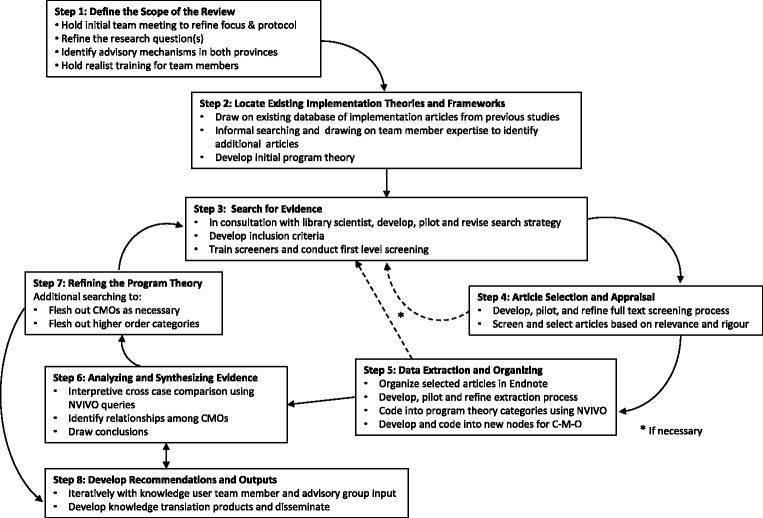
Flow diagram of steps in implementation process. Adapted from Wong G, Brennan N, Mattick K, et al. Interventions to improve antimicrobial prescribing of doctors in training: the IMPACT (IMProving Antimicrobial presCribing of doctors in Training) realist review. BMJ Open 2015;5:e009059. doi:10.1136/bmjopen-2015-009059

In this study, we will follow established realist synthesis quality standards and publication guidelines [26, 30, 31] which lay out a series of steps in an iterative process. A flow diagram of the steps in our proposed realist review process is outlined in Fig. 1 and each step is described below. Ethical approval for the study is not required because no primary data collection is involved. PROSPERO registration has been obtained (CRD42015030052). We completed the PRISMA-P (Preferred Reporting Items for Systematic Review and Meta-Analysis Protocols) checklist [29] (see Additional file 1). Note that we wrote this paper after some of the early steps in the protocol were already initiated or completed. Thus, it was necessary to use both past tense (describing elements that have been completed) and future tense (describing elements that have not yet been initiated).

#### Step 1: define the scope of the review

##### Initial meeting to refine scope and protocol

When notification of funding was received, we brought the entire research team (composed of researchers and knowledge users) together to define the scope and focus of the review, refine our protocol including the research questions, and clarify our approach for working together. This is an integral part of the review process because stakeholder engagement is central to realist review. With respect to refining the protocol, this will continue to evolve as the review progresses wherein we will be guided by our initial and evolving program theory and the results of our search (as described below). Overall, the purpose of our realist review is to explain the implementation process and outcomes for public health interventions within diverse contexts. This will take place iteratively over time as reflected in Fig. 1.

During this initial meeting, the team confirmed that by *public health interventions* we mean *system-wide policies*, *programs*, or *strategies initiated in local*, *regional*, or *state/provincial public health systems*. These interventions aim to improve population health or reduce health inequities. By implementation we mean putting an intervention into action. Although there are many studies of public health interventions, our preliminary informal scoping suggested that few are actually studies of implementation; that is, examination of the process, outcomes, and factors influencing implementation of public health interventions. To ensure an adequate number of studies for our review, we initially focussed our search quite broadly on interventions being implemented in any area of public health, as defined in the BC Core Public Health Functions framework [28] and the Ontario Public Health Standards [29]. Our intent was that if our initial search resulted in an unmanageable number of studies (over 7500), we would narrow our focus to implementation studies focussing on current public health priority intervention areas shared by both provinces as outlined in their new strategic plans [32, 33]: healthy child development; healthy eating, physical activity, tobacco control, and alcohol use; communicable disease prevention; injury prevention; and healthy environments. We have now conducted the initial search in which we identified 5386 papers so we will not be narrowing the focus to the above identified areas.

##### Refine the research questions

We began with a broad question: Why are some public health interventions successfully implemented and others not? As a team, we elaborated on the research question in keeping with the progressive focussing intent of realist synthesis. More specific questions were identified:

1. What are the mechanisms inherent in successful strategies supporting effective implementation (as defined in our initial program theory) of public health interventions?
2. What are the contexts, circumstances, and conditions within which different mechanisms produce different levels of success in implementing public health interventions?
3. What implementation outcomes are considered successful and how is success defined?

##### Our approach for working together

The composition of our team and our way of working together reflects an *integrated knowledge translation* approach [34, 35] defined as involvement of knowledge users at every stage of the research process. This is a collaborative and participatory process for undertaking the review. In our approach, both knowledge users and academic researchers are full members of the research team, unlike many reviews in which stakeholders are external to the team and to the review process. Our knowledge users are active participants in the process and outcome of the review. They represent the national Public Health Agency of Canada, the health ministries and health authorities/health units in British Colombia and Ontario as well as Public Health Ontario—a provincial level organization that links practitioners and researchers to ‘scientific intelligence’, that is knowledge and evidence derived from research. Knowledge users, in addition to being members of the team, serve an important internal advisory mechanism because they have knowledge about and responsibility for implementing public health interventions in their jurisdictions.

In addition to active participation of our knowledge user partners, we will establish relevant advisory groups in each province comprising individuals who know the challenges of implementing public health interventions and have responsibility for implementing the new public health strategic plans in their provinces. We will engage these advisors when we have results to report. Each will bring a different perspective on the findings, the evolving realist program theory, and its refinement. They, along with our knowledge user partners, will advise on appropriate knowledge translation plans, products, and the best methods for dissemination.

##### Implement realist synthesis training for the team

Although some team members have had experience conducting a realist synthesis, the methodology was new for many of us. We therefore held a 2-day training workshop early in the study which was conducted by Wong, our team member with expertise in the methodology. It was attended by our academic researchers, staff members, graduate students, and knowledge users. Additional brief training sessions will be held as necessary at various stages during the review for members of the team.

#### Step 2: locate existing implementation theories and frameworks

The main aim in this step is to identify or develop an initial program theory to guide the search and synthesis process. It will be revised and ultimately transformed into a realist middle-range theory that explains successful implementation of public health interventions through the identification of CMOs and their configurations (CMOCs).

##### Draw on existing database of implementation articles

We began by exploring our existing collection of a broad range of implementation papers gathered over 7 years of conducting various implementation studies of public health policy interventions including the following: (1) implementation of a provincial school-based substance misuse prevention program; (2) implementation of evidence reviews for food safety, food security, unintentional injury prevention, healthy living, and sexually transmitted infection programs; (3) application of an equity lens for the public health programs mentioned in item 2 above as well as in substance use prevention, harm reduction, and mental health promotion programs. Some of these references were gathered in systematic searches conducted for specific studies; others were identified and gathered by various members of our research team as they were writing research proposals or papers for publication. These included articles on implementation theory, implementation frameworks, and studies on the implementation of the specific public health interventions.

##### Conduct informal searching and use team member expertise

We conducted informal searches by (1) hand searching relevant journals (e.g., Implementation Science), (2) identifying papers in the reference lists of the articles in our existing database, (3) conducting quick Google and Google scholar searches using the keywords ‘implementation’ and ‘public heath’, and (4) asking team members to suggest relevant papers based on their own expertise and experience. From these sources, we identified ten implementation theories or frameworks [4, 13, 14, 36–42] that we believed had potential to serve as an initial program theory. Unfortunately, none of them were specific to public health.

##### Develop an initial program theory

In consultation with our researcher and knowledge user team members, we reviewed the various frameworks identified in the steps above and ultimately selected the Consolidated Framework for Implementation Research (CFIR) [36] as the most comprehensive theoretical framework to serve as our initial program theory. The framework incorporates constructs from 19 other implementation theories and frameworks. Although the CFIR is more of a framework than a theory, we believe it provides the main building blocks for constructing a realist program theory. Using a realist ‘lens’, many of its elements, although not specified as such, could be readily conceptualized as a context, mechanism, or outcome. Other elements will need to be elaborated and further analyses will be needed in some cases to build CMOCs. This will take place throughout the review process.

We tested our initial program theory against a small sample of empirical studies that examined the implementation of a system-wide public health policy or program. Using NVivo 10 [43], a qualitative software package, we constructed a coding framework based on the CFIR in which each node represented one CFIR element or construct. We coded data against the framework and entered the text representing each construct into the relevant NVivo node. This process confirmed the framework’s general applicability for understanding implementation of public health interventions. As predicted, however, we identified several important gaps in the CFIR framework as applied to the implementation of public health interventions. Thus, we adapted the framework by adding constructs that the initial test articles indicated were important for understanding implementation of public health interventions. We will make ongoing revisions as we go through the extraction, analysis, and synthesis process, particularly in terms of fleshing out the specific CMOs and their configurations.

Given the lack of implementation frameworks in the literature that are specific to public health, we believe our initial program theory constitutes an important contribution in its own right. We are preparing a second paper for publication on our adaptation of the CFIR for public health applications that will detail the new constructs and their definitions.

#### Step 3: searching for evidence

##### Develop search strategy in consultation with library scientist

A realist synthesis approach to searching for evidence is purposive and iterative and evolves as understanding of the subject matter deepens. Guided by our initial program theory, our aim was to search for empirical studies and theoretical literature to provide data that are relevant to the review questions, related to aspects of the initial program theory, and ‘able to shed light on any aspect of C, M, or O for any element of the theory’ [26].

In consultation with our library scientist, we developed, piloted, and revised an initial search strategy. Using the databases CINAHL, Medline, ERIC, Psyc, and Cochrane (all on Ebscohost platform), as well as Google Scholar and Web of Science, our search specialist developed and tested several strategies to identify one with adequate sensitivity and specificity. The final search strategy is presented in Additional file 2. We restricted our search to papers published in English in the year 2000 or later and from a range of North American and European countries, as well as New Zealand and Australia, that we believed would be most relevant to a range of public health systems. We used the BC Core Public Health Functions Framework [28] which encompassed the same content as the Ontario Public Health Standards [29] to define public health interventions that we would include in the search strategy (see item 4 in Table 1 below). Using this search strategy, 5386 articles were identified.

**Table 1:** Inclusion criteria

1. The paper was published in 2000 or later AND;
2. The paper was published in English AND;
3. The study is from one of the countries of Australia, Canada, Denmark, Finland, Ireland, the Netherlands, New Zealand, Norway, Sweden, Switzerland, the United Kingdom, and the USA AND;
4. The paper is about a public health intervention either:
a. Targeting at least one area of public health: health improvement; disease, injury, or disability prevention; environmental health; health emergency management; or health equity and determinants of health; and employing at least one public health strategy: health promotion; health protection, preventive interventions; or health assessment and disease surveillance OR;
b. Aiming to improve system capacity by providing supportive infrastructure for implementation (research, performance management, information systems, adequate and well-trained human resources) AND;
5. The paper is about a public health policy or program that has been implemented or an implementation intervention that has been implemented AND;
6. The paper includes any of the following in the abstract:
a. The study or discussion of implementation as a specific aim AND/OR;
b. Factors that influence the implementation process or the implementation intervention AND/OR;
c. Implementation outcomes AND/OR;
d. The influence of context on implementation.

##### Develop inclusion criteria

The research team developed the inclusion criteria for the initial selection of articles for review. These criteria are outlined in Table 1 and are the basis for the first-level screening of article titles, abstracts, and keywords. In our pilot testing of these criteria, we realized that there was some confusion by reviewers about whether they should include studies in which there was an implementation plan but the implementation had not actually occurred. For this reason, we added criterion five which specifies that the intervention (either the public health intervention itself, or an implementation intervention) actually had to be implemented for it to be included. Because our search strategy had to strike the balance between sensitivity and specificity, many articles were identified that had the term implementation in the title, abstract, or keywords, but did not actually provide the information required, so criterion 6 was added to improve specificity.

##### Train screeners and conduct first-level screening

Three first-level screeners and three of the investigators participated in the screening training. The first 20 articles from the search were screened by all six of the screeners using the criteria in Table 1 to assign a rating to the article: Yes (include), No (do not include), Unsure (not sure if it meets the criteria), or Maybe (not enough information to determine). All articles identified as ‘Yes’ and ‘Maybe’ will be moved forward for full text screening. Those identified as ‘Unsure’ by the first-level screener will be reviewed by the investigator assigned to that screener to determine whether it is a ‘Yes’, ‘No’, or ‘Maybe’. In a group meeting during training for screening, each article was discussed and a consensus rating determined. The criteria were slightly revised based on the discussion to clarify some of the misunderstandings that arose in during the screening training. This process was repeated twice until consensus was reached.

First-level screening will begin with the each of the three screeners being assigned 200 articles to review. One of the three investigators assigned to screening will review 10 % of the articles screened by one of the three primary reviewers or any marked ‘Unsure.’ All six people will meet to discuss this process and determine the rate of agreement. Going forward, the remaining articles will be divided among the three screeners and the process described above will continue. Disagreements will be discussed by the screener and investigator to arrive at a consensus rating. If disagreements persist, the three investigators (who include two of the principal investigators) will have a discussion to resolve the discrepancy.

#### Step 4: article selection and appraisal

##### Develop, pilot, and refine full text screening

In the pilot testing and training for our first-level screening process, we recognized that it was difficult to determine from the title, abstract, and keywords whether the public health intervention was actually a system-wide policy or program so this will be the first criterion for the full text screening. The same criteria identified in Table 1 will be applied in full text screening, but we anticipate that additional issues may arise in first-level screening that will need to be taken into account during full text screening. Additional criteria will be developed, if necessary, after first-level of screening is completed. We may also find that articles meeting the inclusion criteria in first-level screening may not actually contain sufficient data to further refine the initial program theory—and may need to be excluded at this point. This process will again be pilot tested and screeners will be trained.

##### Select articles based on relevance and rigour

In selecting and appraising the references to be included, the criteria of relevance and rigour are used. The identification of what is relevant to include will be made based on the inclusion criteria and guided by our program theory. A reference is relevant if it can contribute to developing, testing, or refining our initial program theory or parts of it. Decisions about relevance are made before decisions about rigour. It may be necessary to conduct additional searches at this point if there are few articles addressing some of the important aspects of the theory, although a preliminary scan of the references obtained suggests that we should have sufficient data.

A study is rigorous if the methods used to obtain the relevant data are trustworthy and credible [21]. In realist review, the rigour issue revolves around how much we can trust the data we are using to draw inferences, based on how it was generated. In a given document, different data may be relevant to different aspects of the review thus serving different purposes. Therefore, it makes no sense in realist review to use standard checklists to make judgements about overall study rigour because a particular checklist may be appropriate only for a small part of the relevant data in a paper. Also, for other data in the same paper, there may not be an appropriate checklist available.

In general, ‘appraisals of rigour judge the plausibility and coherence of the methods that were used to generate the data’ ([26], p.35) and this judgement might need to be made separately for different data from the same paper. We will follow the recommendation in the RAMESES training materials [26] that for each type of relevant evidence identified, reviewers will identify and make notes about any issues that might affect data quality or rigour. For those papers in which there are questions about quality, the issues will be discussed between the staff member doing the appraisal and the investigator assigned to that reviewer. These judgements will be taken into account in refining the program theory. The most important judgement to be made about data quality in realist synthesis relates to its contribution to the probative value of the program theory. Whether the theory is convincing may not depend solely on the rigour of the data because often circumstantial data from less rigorous studies will still be useful in a convincing theory.

Training will be held for reviewers conducting the assessments of relevance and rigour and pilot tested. Again, a 10 % sample of papers selected by the reviewers will be checked by the investigators assigned to each reviewer and disagreements will be resolved through discussion between the reviewer and the assigned investigator. Unresolved disagreements will be discussed by the three investigators to make a decision.

One of the proposed knowledge translation strategies identified as important by our knowledge user partners was to provide interim recommendations based on our initial program theory and our understanding of the literature as we were beginning to explore it. It is most likely to be at this point in the screening process, after going through full text review and appraisal, that we will be able to make some initial recommendations. We will convene a full team meeting at this point to convey these to our knowledge user partners, with the caveat that there may be changes in the final recommendations.

#### Step 5: data extraction and organizing findings

##### Organize the articles in Endnote

Articles selected for review will be managed in Endnote to support our analysis. For each article, notes will be entered into Endnote to identify the inclusion decision, who reviewed the article, and the category of the article. For example, we may include all articles related to policy interventions in one category and those related to programs in another. We may also categorize by public health area (communicable diseases, environmental health, and injury prevention for example). Being able to review a group of articles on the same topic or on the same type of implementation intervention will facilitate greater familiarity with the data thereby ensuring consistency in coding during the extraction process. At this point, we can map the scope of the literature on implementation of public health interventions. This will be a useful study output in its own right.

##### Develop, pilot, and refine extraction process

The steps of realist synthesis are non-linear, and the processes involved in extraction, analysis, and synthesis are interrelated and iterative. Although we have separated the step of data extraction from analysis and synthesis for convenience in reporting, we recognize that these processes are related. Once we have a clear sense of the range of articles selected for review and have categorized them in Endnote, we will have a better idea of how to develop our extraction processes. These will be developed based on the evolving program theory and will be piloted and revised to ensure that they capture relevant data. We will extract data from documents that allow us to understand, for as many aspects of our program theory as possible, how and why the specific implementation outcome has occurred. Extraction will focus first on the initial program theory categories, and then on the questions, What are the generative mechanisms? In what context? For whom? With what outcome? Note that when we refer to outcomes here we mean implementation outcomes as specified in our initial program theory.

##### Code data using program theory categories

Staff involved in both levels of screening will also extract the data. Portions of the article’s text will be selected and coded according to the appropriate high-level construct in the program theory and entered in to NVIVO. Additional training will be provided and initial coding by each coder will be reviewed by the assigned investigator.

##### Develop and code into new nodes for CMOs

Generative mechanisms will need to be identified from existing high-level constructs in the program theory. These will be developed inductively, deductively, and abductively. Coders will work closely with their assigned investigator to ensure that relevant mechanisms are identified from the extracted data and coded appropriately in NVIVO. At this point, if there are insufficient data to identify important elements of the theory, we may need to do more focussed searches. For ease of comparison, data from NVivo coding reports may be moved into tables and spreadsheets.

For all the steps in coding discussed above, a 10 % sample of each coder’s documents will be reviewed by the assigned investigator. Disagreements will be resolved between the coder and investigator. Those that cannot be resolved will be discussed by all coder-investigator teams to achieve consensus.

#### Step 6: analyzing and synthesizing the evidence

##### Interpretive cross-case comparison

Data analysis is driven by the need to make sense of our initial and evolving program theory. When analyzing the findings from included documents, we will use interpretive cross-case comparison [44] to understand and explain how and why observed implementation outcomes (as specified in our initial program theory) have been successful compared with those that have not. Running queries in NVivo to sort the data by relevant categories will support cross-case comparisons. For example, we can run queries to identify which mechanisms are most likely to result in particular implementation outcomes and in which contexts they occur. Other queries will also allow us to identify categories in the theory for which there are limited data and thus require additional focussed searches in step 7 to saturate the categories in the theory and produce our final realist theory of implementation for public health interventions.

##### Identify relationships among CMOs to produce CMOCs

Again, supported by NVivo, we will run compound queries using ‘near content’ in the search to identify text in the extractions that may show relationships among the various CMOs to help us construct the CMOCs. We will strive to understand how context has/has not influenced the outcome patterns reported in the included articles. Using realist logic, we seek to construct CMOCs for the outcome patterns in the more or less successful implementation interventions.

##### Draw conclusions

Based on all of the above steps in the process, we will be able to draw conclusions. We will try to develop iteratively one or more explanatory theories to account for these CMOCs and develop an understanding of how our CMOCs fit with our initial program theory. We will explore whether the CMOCs tell us anything about how we might need to refine our theory.

#### Step 7: refining the program theory

Throughout the review, we will move iteratively between analyzing specific examples (i.e., the individual articles and the data contained within them), refining the overarching program theory, and if necessary doing further iterative searching for data to test particular theories or parts of the theory. If further searching is required to flesh out the CMOs, CMOCs, and the higher order constructs in the program theory, we will need to revisit earlier steps in the process until we are able to build a coherent and plausible ‘final’ realist program theory.

We will meet with our advisory groups at this time to present and obtain input on findings and the revised program theory, discuss conclusions, and plan for knowledge translation. They will be able to identify recommendations and suggest outputs that can be taken into account in step 8.

#### Step 8: develop recommendations and outputs

In our original proposal, in consultation with our knowledge user team members, we identified a number of study outputs, which are listed below. In earlier steps we may have identified additional outputs on the basis of our review process and discussions with knowledge users and advisory groups.

1. Interim recommendations to knowledge user partners on supporting implementation that can be incorporated into ongoing policy and program modifications. We suggested that this would take place in step 4;
2. A theoretical/conceptual framework to guide implementation planning for public health interventions;
3. Theory-based guidelines for developing and applying implementation strategies and supports targeting different types of interventions, organizations, managers, program planners, and practitioners at different levels in the public health system;
4. An inventory of effective implementation strategies in different public health contexts; and
5. Training materials to support implementation based on our findings about effective implementation strategies, including a handbook, webinars, and captivate videos.

In addition to the knowledge translation outputs described above, we plan to publish at least two interim papers. The first will be a paper on our initial program theory, which is a revision of the Consolidated Framework for implementation Research [44] specific to implementing public health interventions. The second will be a paper mapping the scope and focus of the implementation literature in public health. Finally, we will publish one or more papers on the results of our realist synthesis.

## Discussion

Evidence-based public health requires knowledge, not only about effective interventions but also about strategies for successful implementation. Public health interventions are often complex and context-sensitive making knowledge about effective implementation critical to achieve the public health goals of improving population health and promoting health equity. Although there is some literature on the implementation of public health interventions, there is no comprehensive synthesis that encompasses the full range of research outputs. Thus, there is limited information in a useable synthesized form for public health decision makers, program planners, and practitioners.

Ineffective implementation wastes scarce resources and is neither affordable nor sustainable. Travis and colleagues [45] have identified that developing tools to support and ensure effective implementation is one of six essential health stewardship sub-functions of Ministries of Health. This synthesis will provide evidence needed for governments and local public health agencies to identify and support effective strategies for implementing public health policy and program interventions while taking into account the variable context of public health structure and organization.

The need for this realist synthesis was identified and initiated by our knowledge user partners based on challenges they have experienced, or are anticipating, in implementing public health interventions. This entire project reflects an integrated knowledge translation approach [34] using a collaborative process among knowledge users and academic researchers. Conventional approaches to knowledge synthesis such as systematic reviews are often not well suited to more complex public health interventions [17, 19]. Thus, we believe that a realist synthesis is the most appropriate approach to synthesize knowledge about what is necessary to ensure successful implementation of public health interventions. We anticipate that in this synthesis we will generate useable and relevant information for policy makers, program planners, and practitioners that will contribute to better understanding the context and process by which effective implementation of public health interventions can be achieved.

## Additional files

Additional file 1: PRISMA-P (Preferred Reporting Items for Systematic Review and Meta-Analysis Protocols) checklist. PRISMA-P-checklist - Implementation Realist Synthesis. Data type: textual data on items in PRISMA-P checklist. (PDF 384 kb) Additional file 2:
**Initial search strategy.** Initial search strategy for realist synthesis. Data type: textual data describing the initial search strategy for the synthesis. (PDF 184 kb)

## Abbreviations

BC: British Columbia
CMOCs: Context-mechanism-outcome configurations
CMOs: contexts, mechanisms, and outcomes
ON: Ontario
