# Supplementary material for: Supporting successful implementation of public health interventions: protocol for a realist synthesis
Source: Syst Rev. 2016 Apr 7;5:54. doi: 10.1186/s13643-016-0229-1 (PMC4823871; doi:10.1186/s13643-016-0229-1)
Supplement: Additional file 1: — PRISMA-P (Preferred Reporting Items for Systematic Review and Meta-Analysis Protocols) checklist. PRISMA-P-checklist - Implementation Realist Synthesis. Data type: textual data on items in PRISMA-P checklist. (PDF 384 kb) [file 13643_2016_229_MOESM1_ESM.pdf]

Additional File 1

PRISMA-P (Preferred Reporting Items for Systematic review and Meta-Analysis Protocols) 2015 checklist: recommended items to address in a systematic review protocol*

Section and topic	Item No	Checklist item
**ADMINISTRATIVE INFORMATION**		
**Title:**		
**Identification**	1a	Supporting Successful Implementation of Public Health Interventions: Protocol for a Realist Synthesis
**Update**	1b	Not Applicable
**Registration**	2	PROSPERO registration number: CRD42015030052
**Authors:**		
**Contact**	3a	A. ***Corresponding Author:*** Marjorie MacDonald, School of Nursing, University of Victoria, Room A402a, HSD Building, 3800 Finnerty Road Victoria, BC Canada V8P 5C2, marjorie@uvic.ca. This author is also the guarantor of the review.<br>B. Bernadette Pauly, University of Victoria, bpauly@uvic.ca<br>C. Geoff Wong, University of Oxford, grckwong@gmail.com<br>D. Kara Schick-Makaroff, University of Alberta, kara.schickmakaroff@ualberta.ca<br>E. Thea van Roode, University of Victoria, tvanroode@gmail.com<br>F. Heather Wilson Strosher, University of Victoria, hlwilson@uvic.ca<br>G. Anita Kothari, University of Western Ontario, akothari@uwo.ca<br>H. Ruta Valaitis, McMaster University, Valaitis@mcmaster.ca<br>I. Heather Manson, Public Health Ontario, Heather.Manson@oahpp.ca<br>J. Simon Carroll, University of Victoria, scarroll@uvic.ca

		K. Samantha Tong, Fraser Health Authority, Samantha.Tong@fraserhealth.ca<br>L. Victoria Lee, Fraser Health Authority, Victoria.lee@fraserhealth.ca<br>M. Warren O'Briain, BC Ministry of Health, Warren.OBriain@gov.bc.ca<br>N. Karen Dickenson-Smith, FHA, Karen.Dickenson-Smith@fraserhealth.ca<br>O. Megan Ward, Peel Public Health, Megan.ward@peelregion.ca
**Contributions**	3b	Describe contributions of protocol authors and identify the guarantor of the review<br>A. MM: Conceptualized the study, led writing of grant proposal, drafted protocol paper, participated in developing initial program theory, led staff training for screening and coding, supervised screening and coding, conducted quality checks. MM is the guarantor of the review.<br>B. BP: Participated in conceptualizing the study, participated in proposal writing and developing the initial program theory, wrote sections of the draft protocol paper, provided feedback on subsequent drafts and on the funding proposal, co-supervised screening and coding, conducted quality checks.<br>C. GW: Participated in conceptualizing the study, wrote sections of the proposal and the draft protocol paper, reviewed and provided feedback on drafts of the paper, provided methodological guidance throughout.<br>D. KSM: Participated in conceptualizing the study, reviewed and provided feedback on the draft of the protocol paper and funding proposal; co-supervised screening and coding, conducted quality checks.<br>E. TvR: Managed the overall study, participated in revising the protocol and developing initial program theory, reviewed and provided feedback on the draft of the protocol paper, participated in screening and coding.<br>F. HWS: Participated in conceptualizing the study and drafting the grant proposal, contributed to first draft of protocol paper, reviewed and provided feedback on the draft of the paper, coordinated all team communications, participated in developing the initial program theory.<br>G. AK: Participated in conceptualizing the study and in in developing the initial program theory, reviewed and provided feedback on the draft of the protocol

		paper and funding proposal<br>H. RV: Participated in conceptualizing the study and in in developing the initial program theory, reviewed and provided feedback on the draft of the protocol paper and funding proposal<br>I. HM: Participated in conceptualizing the study and in in developing the initial program theory, reviewed and provided feedback on the draft of the protocol paper and funding proposal<br>J. SC: Participated in conceptualizing the study, wrote a section of the grant proposal, assisted in the realist synthesis training, provided feedback on the draft of the protocol paper and funding proposal.<br>K. ST: reviewed and provided feedback on the draft of the protocol paper, participated in developing initial program theory, participated in realist training, reviewed and provided feedback on protocol paper.<br>L. VL: reviewed and provided feedback on the draft of the protocol paper and funding proposal, provided input on initial program theory.<br>M. WO: participated in conceptualizing the study, reviewed and provided feedback on the draft of the protocol paper and funding proposal<br>N. KDS: reviewed and provided feedback on the draft of the protocol paper and the finding proposal.<br>O. Megan Ward: reviewed and provided feedback on the draft of the protocol paper and funding proposal
**Amendments**	4	If the protocol represents an amendment of a previously completed or published protocol, identify as such and list changes; otherwise, state plan for documenting important protocol amendments<br>NOT APPLICABLE
**Support:**		
**Sources**	5a	Canadian Institutes of Health Research, Knowledge Translation Unit provided funding under grant number: FRN# KRS 138213

**Sponsor**	5b	Same as 5a above
**Role of sponsor or funder**	5c	The funder had no role in developing the proposal or conducting the study.
**INTRODUCTION**		
**Rationale**	6	There is a growing emphasis in public health on the importance of evidence-based interventions to improve population health and reduce health inequities. Equally important is the need for knowledge about how to implement these interventions successfully. Yet, a gap remains between the development of evidence-based public health interventions and their successful implementation. Traditional systematic reviews have been conducted on effective implementation in health care, but few in public health so their relevance to public health is unclear. In most reviews, stringent inclusion criteria have excluded entire bodies of evidence that may be relevant for policy makers, program planners, and practitioners to understand implementation in the unique public health context. Realist synthesis is a theory-driven methodology that draws on diverse data from different study designs to explain how and why observed outcomes occur in different contexts and thus may be more appropriate for public health.
**Objectives**	7	This realist synthesis addresses the following overarching question: Why are some public health interventions implemented successfully and others not? More specific questions are: (1) What are the mechanisms inherent in successful strategies supporting effective implementation (as defined in our initial program theory) of public health interventions? (2) What are the contexts, circumstances, and conditions within which different mechanisms produce different levels of success in implementing public health interventions? (3) What implementation outcomes are considered successful and how is success defined?<br><br>Participants include public health decision makers, program planners and practitioners responsible for developing and implementing complex public health interventions in local, regional, state/provincial public health systems. Public health interventions are defined system-wide policies, programs, or strategies initiated in local, regional or

		state/provincial public health systems. They aim to improve population health and/or reduce health inequities. We will be comparing the contexts, mechanisms, and outcomes of successfully implemented public health interventions with those that are not successfully implemented. We will also be comparing different types of public health interventions in terms of their contexts, underlying mechanisms and implementation outcomes.<br><br>The outcomes of concern are not the anticipated health related outcomes of the public health programs and policies, but the outcomes of strategies to ensure that public health interventions are implemented successfully. The outcomes were will be looking for are staged, and include: awareness of the intervention; adoption of the intervention by an individual practitioner or decision maker, or by an organization; implementation of the intervention with fidelity to the plan; penetration of the intervention in the organization and its subsystems; sustainability or maintenance of the intervention over time; and level/extent to which implementation has been achieved. We anticipate that in the course of the review we will identify additional implementation outcomes.<br><br>The objectives of our study are to:<br>1) To understand the contexts and mechanisms that influence the degree to which system-wide public health policies and programs are implemented.<br>2) To determine whether current implementation frameworks are adequate for public health at the population level.<br>3) To contribute to the development of the realist review methodology for public health interventions.<br>4) Develop a series of knowledge translation products that will be helpful to our knowledge user partners in supporting implementation of public health interventions in their organizations and beyond.
**METHODS**		
**Eligibility criteria**	8	Inclusion criteria for selection of articles:<br><br>1. The paper was published in 2000 or later AND

		2. The paper was published in English AND<br>3. The study is from one of the countries of: Australia, Canada, Denmark, Finland, Ireland, the Netherlands, New Zealand, Norway, Sweden, Switzerland, the United Kingdom, the United States AND<br>4. The paper is about a public health intervention either:<br>a. Targeting at least one area of public health: health improvement; disease, injury or disability prevention; environmental health; health emergency management; or health equity and determinants of health; and employing at least one public health strategy: health promotion, health protection, preventive interventions, or health assessment and disease surveillance OR<br>b. Aiming to improve public health system capacity by providing supportive infrastructure for implementation (research, performance management, information systems, adequate and well trained human resources) AND<br>5. The paper is about a public health policy or program that has been implemented OR an implementation intervention that has been implemented AND<br>6. The paper includes discussion of any of the following in the abstract:<br>a. the study of implementation is a specific aim OR<br>b. describes factors influencing implementation process/implementation intervention OR<br>c. implementation outcomes OR<br>d. the influence of context on implementation.
**Information sources**	9	Using the databases CINAHL, Medline, ERIC, Psyc, Cochrane (all on Ebscohost platform), as well as Google Scholar and Web of Science, our search specialist developed and tested several strategies to identify one with adequate sensitivity and specificity. We restricted our search to papers published in English in the year 2000 or later and from a range of North American and European countries that we believe will be most relevant to a range of public health systems (see number 3 above in eligibility criteria).

**Search strategy**	10	Databases: CINAHL, Medline, ERIC, Psyc, Cochrane (all on Ebscohost platform)<br>1. (Denmark or Finland or Norway or Sweden or Switzerland or Netherlands or Ireland or United kingdom or Great Britain or Wales or Scotland or England or Australia or Canada or New Zealand or United States) (searched as subject word in Cinahl and Medline; place location in Psyc; keyword in Eric and Cochrane)<br>2. (British Columbia or Alberta or Saskatchewan or Manitoba or Ontario or Quebec or New Brunswick or Nova Scotia or Newfoundland or Nunavut or PEI or Prince Edward Island or Labrador or NWT or Northwest Territories or Yukon) (searched in title word, subject word and abstract fields)<br>3. (Set 1 or Set 2)<br>4. ("public health" or "health promotion" or "disease prevention" or "primary prevention" or "injury prevention" or "chronic disease prevention" or "population health" or "population health intervention*" or "public health intervention*" or "health equity”) (searched in title word, subject word and abstract fields)<br>5. implement*(searched in title word and subject word fields) OR implementation (searched in abstract field)<br>6. ("knowledge transfer" or "knowledge translation" or "translational medical research" or implement* n2 program* or implement* n2 policy or implement* n2 strateg* or implement* n2 health or implement* n2 intervention*) (searched in abstract field)<br>7. ("knowledge transfer" or "knowledge translation" or "translational medical research") (searched in title word and subject word fields)<br>8. Set 5 or Set 6 or Set 7<br>9. Set 3 and Set 4 and Set 8<br>10. Limited to January 1, 2000 onwards<br>11. Limited to English language publications

		Database: Google Scholar.<br><br>Each of the concepts listed in set 4 was searched separately as keywords, combining them with the concepts of implementation or “knowledge translation” or “knowledge transfer” as title words, and the publication date of 2000 onwards, and then downloaded the first 30 hits from each search, excluding those outside the country parameters.<br><br>Database: Web of Science:<br><br>1. (Denmark or Finland or Norway or Sweden or Switzerland or Netherlands or Ireland or United kingdom or Great Britain or Wales or Scotland or England or Australia or Canada or New Zealand or United States) (topic search)<br>2. ("public health" or "health promotion" or "disease prevention" or "primary prevention" or "injury prevention" or "chronic disease prevention" or "population health" or "population health intervention*" or "public health intervention*" or "health equity”) (topic search)<br>3. ("knowledge transfer" or "knowledge translation" or "translational medical research" or implement*) (title search)<br>4. Set 1 and Set 2 and Set 3<br>5. Limited to January 1, 2000 onwards<br>6. Limited to English language publications
**Study records:**		
**Data management**	11a	Articles selected for review will be managed in Endnote where we can track the reviews, who has reviewed them, and categorize them into subgroups that may help to manage the analysis. For example, we may include all articles related to policy interventions in one category and those related to programs in another. We may also categorize by the public health area (communicable diseases, environmental health, and injury prevention for example). It may help the person extracting data in the next stage to be able to review a group of articles on the same topic or on the same type of implementation intervention to allow the reviewer to become more familiar with the data and thus

		achieve more consistency in coding. At this point we can map the scope of the literature we have gathered to get a clear understanding of the literature base on implementation of public health interventions.<br><br>The full texts of selected papers will then be imported into NVivo 10 (a qualitative data analysis package) for data extraction and analysis.
	11b	Three first level screeners and three of the investigators participated in training for screening. The first 20 articles from the search were screened by all six of the screeners using the inclusion criteria specified in item 8 to assign a rating to the article: Yes (include), No (do not include), Unsure (not sure if it meets the criteria) or Maybe (not enough information to determine). Articles identified as 'Maybe' will be moved forward for full text screening. Those identified as 'Unsure' by the first level screener will be reviewed by the investigator assigned to that screener. In a group meeting, each article was discussed and a consensus rating determined. The criteria were slightly revised based on the discussion to clarify some of the misunderstandings that arose in the screening process. This process was repeated twice until consensus was reached.<br><br>First level screening will begin with the each of the three screeners being assigned 200 articles to review. One of the three investigators assigned to screening will review 10% of the articles screened by one of the three primary reviewers or any marked 'Unsure.' All six people will meet to discuss this process and determine the rate of agreement. Going forward, the remaining articles will be divided among the three screeners and the process described above will continue. Disagreements will be discussed by the screener and investigator to arrive at a consensus rating. If disagreements persist, the three investigators (who include two of the principal investigators) will have a discussion to resolve the discrepancy. All articles selected in this first level screening will be subject to a full text screening.<br><br>Because it was difficult to determine from the title, abstract and key words in the first level screening whether the public health intervention was actually a system-wide policy or program, this will be the first criterion for the full text screening. The same criteria identified in item 8 will be applied in full text screening, but we anticipate that additional

		issues may arise in first level screening that will need to be taken into account during full text screening. This process will again be pilot tested and screeners will be trained.<br><br>In selecting and appraising the studies to be included, the criteria of relevance and rigour are used. Relevance is based on the inclusion criteria, and guided by our program theory. A reference is relevant if it can contribute to developing, testing or refining our initial program theory or parts of it. Decisions about relevance are made before decisions about rigour. A study is rigorous if the methods used to obtain the relevant data are trustworthy and credible. In a given document, different data may be relevant to different aspects of the review thus serving different purposes. Therefore it makes no sense in realist review to use standard checklists to make judgements about overall study rigour because a particular checklist may be appropriate only for a small part of the relevant data in a paper. Also, for other data in the same paper there may not be an appropriate checklist available.<br><br>In general, judgements of rigour might need to be made separately for different data from the same paper. We will follow the recommendation in the RAMESES realist synthesis training materials that for each type of relevant evidence identified, reviewers will identify and make notes about any issues that might affect data quality or rigour. For those papers in which there are questions about quality, the issues will be discussed between the staff member doing the appraisal and the investigator assigned to that reviewer. These judgements will be taken into account in refining the program theory. The most important judgement to be made about data quality in realist synthesis relates to its contribution to the probative value of the program theory. Whether the theory is convincing may not depend solely on the rigour of the data because often circumstantial data from less rigorous studies will still be useful in a convincing theory.<br>Training will be held for reviewers conducting the assessments of relevance and rigour and pilot tested. Again, a 10% sample of papers selected by the reviewers will be checked by the investigators assigned to each reviewer and disagreements will be resolved through discussion between the reviewer and the assigned investigator. Unresolved disagreements will be discussed by the three investigators to make a decision
**Data collection process**	11c	Once we have a clear sense of the range of articles selected for review and have

categorized them in Endnote, we will have a better idea of how to develop our extraction processes. These will be developed based on the evolving program theory and will be piloted and revised to ensure that they capture relevant data. We will extract data from documents that allow us to understand, for as many aspects of our program theory as possible, how and why the specific implementation outcome has occurred. Extraction will focus first on the initial program theory categories, and then on the questions: What are the generative mechanisms? In what context? For whom? With what outcome? Note that when we refer to outcomes here we mean implementation outcomes as specified in our initial program theory.

Staff involved in both levels of screening will also extract the data. Portions of the article's text will be selected and coded into the appropriate high level construct in the program theory using NVIVO 10 qualitative software [50]. Additional training will be provided and initial coding by each coder will be reviewed by the assigned investigator. Generative mechanisms will need to be identified from existing high level constructs in the program theory. These will be developed inductively, deductively and abductively. Coders will work closely with their assigned investigator to ensure that relevant mechanisms are identified from the extracted data and coded appropriately in NVIVO. For all the steps in coding discussed above, a 10% sample of each coder's documents will be reviewed by the assigned investigator. Disagreements will be resolved between the coder and investigator. Those that cannot be resolved will be discussed by all coder-investigator teams to achieve consensus.

The full texts of the selected papers will first coded with attributes to allow use of the query function in NVivo to determine differences by various categories of the papers (e.g., type of public health intervention, type of public health practitioner involved, type of intervention strategy, and other categories to be identified after initial screening). Coding will be initially deductive, guided by the initial program theory categories which have been pre-defined. This will be followed by inductive coding of data within the text that identifies specific mechanisms that are operating in the intervention strategy, the contexts in which those mechanisms fire, and the outcomes that are achieved. As well, we will be coding the relationships among contexts, mechanisms and outcomes. The extracted data will be analyzed to refine the initial program theory if necessary. As the

		theory is revised and refined, the coded studies will be re-assessed and examined to find data that can help to refine and elaborate the theory. New searches may be required to find additional data that can aid in this process.
**Data items**	12	
**Outcomes and prioritization**	13	As noted above, portions of the article's text will be selected and coded into the appropriate high level construct in the program theory using NVIVO 10 qualitative software. Our initial program theory is very complex with multiple variables too numerous to list here. They can be grouped, however, into the categories of: Public Health Intervention Characteristics; Implementation Intervention Characteristics; Outer Setting (or larger system context); Inner Setting (or organizational context); Characteristics of the Implementers; Community Characteristics; Implementation Process; Implementation Outcomes.<br><br>Once the coding is completed for the step described above, generative mechanisms will need to be identified from existing high level constructs in the program theory. These will be developed inductively, deductively and abductively. Coders will work closely with their assigned investigator to ensure that relevant mechanisms are identified from the extracted data and coded appropriately in NVIVO. At this point, if there are insufficient data to identify important elements of the theory, we may need to do more focussed searches. For ease of comparison, data from NVIVO coding reports may be moved into tables and spreadsheets.
**Risk of bias in individual studies**	14	In selecting and appraising the references to be included, the criteria of relevance and rigour are used. A reference is relevant if it can contribute to developing, testing or refining our initial program theory or parts of it. Decisions about relevance are made before decisions about rigour. A study is rigorous if the methods used to obtain the relevant data are trustworthy and credible. In a given document, different data may be relevant to different aspects of the review thus serving different purposes. Therefore it makes no sense in realist review to use standard checklists to make judgements about overall study rigour because a particular checklist may be appropriate only for a small part of the relevant data in a paper. Also, for other data in the same paper there may not be an

		appropriate checklist available.<br><br>In general, “appraisals of rigour judge the plausibility and coherence of the methods that were used to generate the data” and this judgement might need to be made separately for different data from the same paper. We will follow the recommendation in the RAMESES training materials that for each type of relevant evidence identified, reviewers will identify and make notes about any issues that might affect data quality or rigour. For those papers in which there are questions about quality, the issues will be discussed between the staff member doing the appraisal and the investigator assigned to that reviewer. These judgements will be taken into account in refining the program theory. The most important judgement to be made about data quality in realist synthesis relates to its contribution to the probative value of the program theory. Whether the theory is convincing may not depend solely on the rigour of the data because often circumstantial data from less rigorous studies will still be useful in a convincing theory.<br><br>Training will be held for reviewers conducting the assessments of relevance and rigour and pilot tested. A 10% sample of papers selected by the reviewers will be checked by the investigators assigned to each reviewer and disagreements will be resolved through discussion between the reviewer and the assigned investigator. Unresolved disagreements will be discussed by the three investigators to make a decision.
**Data synthesis**	15a	Data analysis and synthesis is driven by the need to make sense of our initial and evolving program theory. When analyzing the findings from included documents, we will use interpretive cross case comparison to understand and explain how and why observed outcomes have been successful compared with those that have not. We will be aided in this cross case comparison by running queries in NVIVO which will sort the data by relevant categories. For example, we can run queries to identify which mechanisms are most likely to result in particular outcomes and in which contexts they occur. Queries will

		also allow us to identify categories in the theory for which there are limited data and thus require additional focussed searches to saturate the categories in the theory and produce our final realist theory of implementation for public health interventions.<br><br>Again, supported by NVIVO, we will run compound queries using "near content" in the search to identify text in the extractions that may show relationships among the various contexts, mechanisms and outcomes (CMO) to help us construct the configurations and relationships among these (CMOCs). We will strive to understand how context has/has not influenced the outcome patterns reported in the included articles. Using realist logic, we seek to construct CMOCs for the outcome patterns in the more or less successful implementation interventions.
	15b	N/A
	15c	N/A
	15d	Based on all of the steps in the process, we will be able to draw conclusions. We will iteratively develop one or more explanatory theories to account for the CMOCs and develop an understanding of how our CMOCs fit with our initial program theory. We will explore whether the CMOCs tell us anything about how we might need to refine our theory.
**Meta-bias(es)**	16	Not applicable for realist synthesis
**Confidence in cumulative evidence**	17	Not applicable for realist synthesis
