# Supplementary material for: Supporting successful implementation of public health interventions: protocol for a realist synthesis
Source: Syst Rev. 2016 Apr 7;5:54. doi: 10.1186/s13643-016-0229-1 (PMC4823871; doi:10.1186/s13643-016-0229-1)
Supplement: Additional file 2: — Initial search strategy. Initial search strategy for realist synthesis. Data type: textual data describing the initial search strategy for the synthesis. (PDF 184 kb) [file 13643_2016_229_MOESM2_ESM.pdf]

Additional File 2
Initial Search Strategy for Realist Synthesis on
Implementation of Public Health Interventions

Returned: 5386 articles

Databases: CINAHL, Medline, ERIC, Psyc, Cochrane (all on Ebscohost platform)

1. (Denmark or Finland or Norway or Sweden or Switzerland or Netherlands or Ireland or United kingdom or Great Britain or Wales or Scotland or England or Australia or Canada or New Zealand or United States) (searched as subject word in Cinahl and Medline; place location in Psyc; keyword in Eric and Cochrane)
2. (British Columbia or Alberta or Saskatchewan or Manitoba or Ontario or Quebec or New Brunswick or Nova Scotia or Newfoundland or Nunavut or PEI or Prince Edward Island or Labrador or NWT or Northwest Territories or Yukon) (searched in title word, subject word and abstract fields)
3. (Set 1 or Set 2)
4. ("public health" or "health promotion" or "disease prevention" or "primary prevention" or "injury prevention" or "chronic disease prevention" or "population health" or "population health intervention*" or "public health intervention*" or "health equity") (searched in title word, subject word and abstract fields)
5. implement*(searched in title word and subject word fields) OR implementation (searched in abstract field)
6. ("knowledge transfer" or "knowledge translation" or "translational medical research" or implement* n2 program* or implement* n2 policy or implement* n2 strateg* or implement* n2 health or implement* n2 intervention*) (searched in abstract field)
7. ("knowledge transfer" or "knowledge translation" or "translational medical research") (searched in title word and subject word fields)
8. Set 5 or Set 6 or Set 7
9. Set 3 and Set 4 and Set 8
10. Limited to January 1, 2000 onwards
11. Limited to English language publications

Database: Google Scholar.

Each of the concepts listed in set 4 was searched separately as keywords, combining them with the concepts of implementation or “knowledge translation” or “knowledge transfer” as title words, and the publication date of 2000 onwards, and then downloaded the first 30 hits from each search, excluding those outside the country parameters.

Database: Web of Science:

1. (Denmark or Finland or Norway or Sweden or Switzerland or Netherlands or Ireland or United kingdom or Great Britain or Wales or Scotland or England or Australia or Canada or New Zealand or United States) (topic search)
2. ("public health" or "health promotion" or "disease prevention" or "primary prevention" or "injury prevention" or "chronic disease prevention" or "population health" or "population health intervention*" or "public health intervention*" or "health equity”) (topic search)
3. ("knowledge transfer" or "knowledge translation" or "translational medical research" or implement*) (title search)
4. Set 1 and Set 2 and Set 3
5. Limited to January 1, 2000 onwards
6. Limited to English language publications
